# Non-invasive messenger RNA transcriptional evaluation in human kidney allograft dysfunction

**DOI:** 10.1590/1414-431X20186904

**Published:** 2018-05-17

**Authors:** G. Joelsons, T. Domenico, L.F. Gonçalves, R.C. Manfro

**Affiliations:** 1 Programa de Pós-Graduação em Medicina: Ciências Médicas, Faculdade de Medicina, Universidade Federal do Rio Grande do Sul, Porto Alegre, RS, Brasil; 2 Serviço de Nefrologia, Hospital de Clínicas de Porto Alegre, Porto Alegre, RS, Brasil

**Keywords:** Kidney transplantation, Acute rejection, Gene expression, Diagnosis, mRNA

## Abstract

The aim of the present study was to evaluate messenger RNA expression in kidney allograft recipients. Forty-four kidney transplant recipients were evaluated up to three months after grafting. After transplantation, peripheral blood samples were drawn sequentially for real-time polymerase chain reaction analyses of perforin and TIM-3 genes. Biopsies were obtained to evaluate acute graft dysfunction and interpreted according to the Banff classification. Eight patients presented episodes of acute rejection. Recipients with rejection had significantly higher levels of TIM-3 mRNA transcripts compared to those without rejection (median gene expression 191.2 and 36.9 mRNA relative units, respectively; P<0.0001). Also, perforin gene expression was higher in patients with rejection (median gene expression 362.0 and 52.8 mRNA relative units; P<0.001). Receiver operating characteristic curves showed that the area under the curve (AUC) for the TIM-3 gene was 0.749 (95%CI: 0.670–0.827). Perforin gene mRNA expression provided an AUC of 0.699 (95%CI: 0.599 to 0.799). Overall accuracy of gene expression was 67.9% for the TIM-3 gene and 63.6% for the perforin gene. Combined accuracy was 76.8%. Negative predictive values were 95.3% for the TIM-3 gene, 95.5% for the perforin gene, and 95.4% in the combined analyses. Gene expression was significantly modulated by rejection treatment decreasing 64.1% (TIM-3) and 90.9% (perforin) compared to the median of pre-rejection samples. In conclusion, the longitudinal approach showed that gene profiling evaluation might be useful in ruling out the diagnosis of acute rejection and perhaps evaluating the efficacy of treatment.

## Introduction

Kidney transplantation has become the therapy of choice for many patients with end-stage renal disease. In the last two decades, significant improvements occurred in the first year post transplantation, but despite this early success, long-term survival of patients and allografts has not improved significantly (1).

Acute rejection (AR), defined as graft aggression resulting from the recipient's immune response to the donor antigens expressed in grafted organs, is a major immunological event and may influence short- and long-term outcomes (2). It typically occurs during the initial periods following renal transplantation and its diagnosis is suspected by an increment in serum creatinine and confirmed by histological analysis of graft tissue. A major difficulty in current clinical practice is that biomarkers presently used in renal transplantation are not accurate enough to differentiate AR from other causes of graft dysfunction, such as acute tubular necrosis, calcineurin inhibitors nephrotoxicity, and infections. No single satisfactory method of diagnosing AR is currently available. Instead, combined methods are used for post-transplantation monitoring.

Histological examination of allograft tissue remains the gold standard for the diagnosis of allograft dysfunction (3). Recent refinements have reduced but not abolished biopsy-associated complications; however, sampling errors, poor reproducibility in interpretation, and the focal characteristic of the inflammatory process of rejection pose additional problems leading to the need of multiple samples to increase diagnostic accuracy (4). Also to be considered are the elevated costs of the biopsy procedure. On the other hand, protocol biopsies may display features of inflammation occurring in well-functioning grafts, the so-called subclinical rejection, that has been associated with chronic graft loss (5).

Gene expression profiling in the early post-transplant period may provide insight into the activity of the immune system in response to the graft (6). Non-invasive tools have several advantages that include the frequent and sequential assessments of the recipient's immune status. The developments in the molecular monitoring of recipients of solid organ transplants have focused on non-invasive tests of easily accessible biological fluids, such as urine and peripheral blood (7–11). Results obtained with hypothesis-driven candidate messenger RNAs (mRNA) expression patterns evaluated in urine and blood have been impressive in cross-sectional studies suggesting that molecular perturbations may precede not only graft dysfunction but also histological changes. Among the studied genes, perforin and TIM-3 have performed highly in terms of diagnostic accuracy in the mentioned cross-sectional studies (8,10,12,13). Perforin is stored in cytoplasmic granules and subsequently secreted by effector CTL leading to pore formation in the target-cell membrane, ultimately leading to cell death (14). TIM-3 is a type I membrane protein preferentially expressed on terminally differentiated Th1 cells, which seems to be central in the mechanisms of allograft rejection (15). However, it must be acknowledged that the vast majority of the studies employing these tools are cross-sectional and that the validation of biomarkers still requires the demonstration of adequate accuracy in longitudinal follow-up studies.

In the present study, we analyzed TIM-3 and perforin mRNA expression in the peripheral blood of kidney transplant recipients to evaluate their utility as non-invasive biomarkers of anti-allograft responses.

## Material and Methods

### Subjects

Forty-four kidney transplant recipients were enrolled. They agreed to participate by signing an informed consent form, and blood samples were sequentially drawn at days 3, 4–6, 9–11, 14–16, 19–21, 24–26, 29–31, 44–46, 59–61, 89–91 after transplantation. Acute rejection was diagnosed by histopathological analysis of graft biopsies or by surveillance biopsies in patients with delayed graft function (DGF). DGF was defined by the need of dialysis within the first week after transplantation. Two cores were obtained at each biopsy and the slides were interpreted according to the Banff classification (3) by a pathologist unaware of the clinical suspicion. Biopsies rated as Banff 1A or higher were considered as rejection. Based on the occurrence of acute rejection, patients were classified as either rejectors or non-rejectors and their gene expression was studied accordingly.

### Immunosuppression and anti-rejection therapy

All patients received a 500-mg dose of methylprednisolone transoperatively and were maintained with a combination of prednisone, sodium mycophenolate, and tacrolimus or cyclosporine. Patients at high-risk for acute rejection received antibody induction therapy with anti-thymocyte antibodies and patients with post-operative oliguria or anuria received anti-IL2 receptor antibodies within 24 h of the transplant surgery. Acute cellular rejections were treated with a 3-day course of 500 mg methylprednisolone intravenously. Steroid-resistant rejections and those with initial classification of Banff IIA or higher were treated with a 10-14-day course of anti-thymocyte antibodies.

### Sample handling and design of primers and probes

Peripheral blood samples were drawn in EDTA-containing tubes and leukocytes were obtained through erythrocyte lysis with a hypotonic buffer and stored at -80°C. RNA isolation was performed using the QiaAmp RNA Blood Mini Kit (Qiagen Inc., USA) according to the manufacturer's instructions. Total RNA quantifications were made using the NanoDrop® 1000 Spectrophotometer v.3.7 (Thermo Fischer Scientific, USA) and RNA purity was observed as a ratio of absorbances at two different wave lengths (260/280 nM). Only samples with optical density ratio higher than 1.7 were analyzed. Total RNA was reverse transcribed into cDNA using the cDNA High Capacity Kit (Applied Biosystems, USA), according to manufacturer's instructions, to a final volume of 20 μL and stored at -20°C.

The 5′ nuclease assay was performed using the ABI 7000 Sequence Detection System and TaqMan Universal PCR Master Mix, composed by AmpliTaq Gold® DNA polymerase, Amperase UNG, passive reference (ROX), buffer and dNTPs (Applied Biosystems, USA). The design and synthesis of the gene specific primers and fluorogenic probes for Perforin (ID: Hs 00169473_m1; GenBank reference: 5551; also listed as PRF1) and TIM-3 (ID: Hs 00262170_m1; GenBank reference: 84868; also listed as HAVCR2) mRNA were made by TaqMan® Gene Expression Assays (Applied Biosystems, USA) and had already been tested and validated previously by the manufacturer. 18S rRNA, was used as an endogenous control (Eukaryotic 18S rRNA Endogenous Control, Applied Biosystems). Gene Expression Assays consisted of 20× concentrated (360 μM) mix of PCR primers and Taqman® MGB (Minor Groove Binding) probes. These assays are designed for the detection and amplification of specific genetic sequences. All primers utilized were intron-spanning to avoid genomic DNA amplification (Gene Expression Assays/Custom Primers and Probes; Applied Biosystems, USA). The Taqman® probes were labeled with FAM (6-carboxyfluorescein) as the reporter at the 5′, except the endogenous control 18S rRNA that was labeled with the dye VIC as the reporter. Gene expression relative quantitation was measured as a rise in fluorescence, resulting from amplification and probe degradation. The cycle in which the fluorescence exceeds the detection threshold is called threshold cycle (Ct). Specific templates in a sample result in an earlier exceeding fluorescence. The sample from the third day post-transplant was used as calibrator. For the diagnosis of rejection and analysis of anti-rejection therapy, the last sample before the beginning of rejection treatment was used. The analyses of amplified products were performed by the relative quantification method 2^-ΔΔCt^, which describes alterations to the target gene expression relative to a reference sample (16).

### Statistical analyses

Descriptive analyses, means±SD, and distributions are reported. Receiver operating characteristic (ROC) curves and non-parametric Mann-Whitney and U-Wilcoxon tests were used for the statistical analysis of quantitative variables. Fisher's exact test was used to compare qualitative variables and non-parametric Wilcoxon signed-rank test was used to compare gene expression levels pre- and post-treatment. ROC curves were generated to analyze diagnostic parameters derived from gene expression. A P level <0.05 was considered for statistical significance. The threshold for overexpression of the genes evaluated (100% relative to the calibrator) were established by ROC curves and used for the calculation of the diagnostic parameters.

The study was approved by the research and ethics committee of Hospital de Clínicas de Porto Alegre and registered at the Office for Human Research Protection.

## Results

Kidney transplant recipients were sequentially evaluated during the initial 90 days after transplantation. Patients were divided according to the occurrence of acute rejection based on graft pathology. The main demographic characteristics of the groups are shown in Table 1. No significant difference was found for the comparisons of gender, age, race, cold ischemia time, human leukocyte antigen (HLA) matching (loci A, B, and DR), incidence of DGF, serum creatinine up to 180 days after transplantation, percentage of deceased donor recipients, and immunosuppressive regimen. The eight rejection episodes were classified as Banff IA (4 episodes), IB (1 episode), IIA (1 episode), and IIB (2 episodes). Biopsies with borderline rejection occurred in 10 patients and were not included in the rejection group.

**Table 1.** Demographic and transplant data of the study subjects.

Parameter	Rejectors (n=8)	Non-rejectors (n=36)	P value
Recipient gender (male/female)^a^	5/3	20/16	0.720
Age (years)^b,c^	46±10	44±13	0.736
Race (Caucasian/non-Caucasian)^d^	6/2	33/3	0.207
Panel reactivity antibodies (%)^b,c^	2.8±7.8	4.6±14.8	0.734
Cold ischemia time (h)^b,c^	17.5±4.3	19.7±6.0	0.399
Donor (living/deceased)^a^	2/6	16/20	0.439
HLA mismatches (A, B, DR)^b,c^	3.2±0.9	2.9±1.4	0.535
DGF cases (%)^a^	6 (75)	13 (36)	0.060
Serum creatinine POD 30 (mg/dL)^b,c^	2.38±1.01	2.05±1.73	0.602
Serum creatinine POD 60 (mg/dL)^b,c^	1.79±0.79	1.76±1.1	0.941
Serum creatinine POD 90 (mg/dL)^b,c^	2.07±0.87	1.64±1.10	0.307
Serum creatinine POD 180 (mg/dL)^b,c^	1.78±0.72	1.83±1.38	0.929
Serum creatinine POD 360 (mg/dL)^b,c^	1.78±0.78	1.86±1.57	0.885
Urinary PCR at 30^th^ POD^b,c^	1.76±0.79	1.71±1.35	0.915
Immunosuppression			
CI+sodium mycophenolate+steroids	4	18	0.487
Induction+CI+sodium mycophenolate+steroids^e^	4	18	

^a^Fisher's exact test; ^b^means±SD; ^c^Student's *t*-test; ^d^Pearson's chi-squared test; ^e^Induction: 4 patients in the rejectors group and 13 patients in the non-rejectors group received Basiliximab®. The others received Thymoglobulin® or calcineurin inhibitors (CI, cyclosporine, or tacrolimus). HLA: human leukocyte antigen; DGF: delayed graft function; POD: post-operative days; PCR: protein/creatinine ratio.

### Gene expression analyses

Eight recipients presented an acute rejection episode. Twenty-eight peripheral blood samples were drawn from these subjects and compared to 243 samples that included all samples from 36 patients without rejection (n=197) and the post-treatment samples of the patients with rejection (n=46).

**Figure 1.**
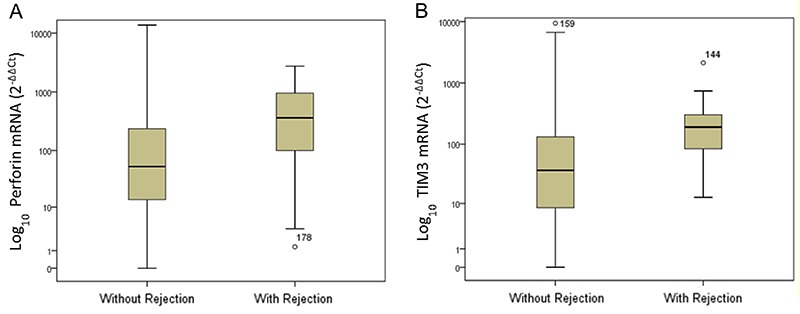
Levels of perforin (*A*) and TIM-3 mRNA (*B*) gene expression in peripheral blood leukocytes of kidney allograft recipients with and without acute rejection. Data are reported as medians, minimum, and maximum values, and 25–75% interquartile range.

Recipients with rejection had significantly higher levels of TIM-3 mRNA transcripts compared to those without rejection. The median gene expression values were 191.2 and 36.9 mRNA relative units, respectively (Mann-Whitney, P<0.001). A significant difference was also observed in the median mRNA expression of the perforin gene: 362 and 52.8 mRNA relative units, respectively, for patients with and without acute rejection (Mann-Whitney; P=0.001). Figure 1 displays the box plots of logarithmic transformed mRNA expressions comparing patients with and without acute rejection.

Box plots show the 10th, 25th, 50th (median), 75th, and 90th percentile values for mRNA gene expression values related to the calibrator (2^-ΔΔCt^) using 18S rRNA as endogenous control. The logarithmic transformed mRNA levels of perforin and TIM-3 were higher in leukocytes from patients with acute rejection than in patients without rejection. Perforin and TIM-3 mRNA levels were higher in the acute-rejection group than in non-rejection group (P<0.001) (Panels A and B).

**Figure 2.**
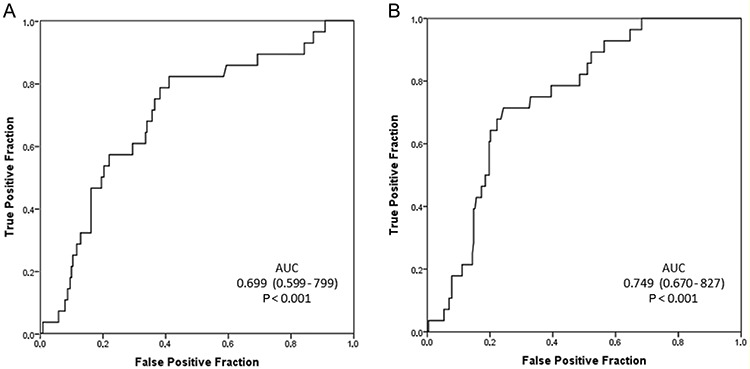
Perforin (*A*) and TIM-3 ROC (*B*) curves of gene expression in the peripheral blood for acute rejection diagnosis of kidney allografts.

ROC curves were generated to analyze the diagnostic parameters of mRNA expression. The areas under the curve (AUC) observed for the TIM-3 and perforin genes are shown in Figure 2. Analyses of expression for both genes resulted in statistically significant AUCs (P<0.001). For TIM-3 gene, the diagnostic parameters were sensitivity of 71.4%, specificity of 67.5%, positive predictive value of 20.2%, and negative predictive value of 95.3% accuracy. Perforin gene diagnostic parameters were sensitivity of 75.0%, specificity of 62.2%, positive predictive value of 18.7%, and negative predictive value of 95.5% accuracy. All 8 episodes of acute rejection presented increased expression of one or both genes. Among the 28 samples obtained in these 8 events, 22 had a raise in the expression in one or both genes (78.6%), 19 had an increased expression in both genes (86.4%), one had isolated increased expression of TIM-3 (4.5%), and 2 samples had isolated increased expression of perforin (9.1%). Combined gene analyses (TIM-3 and perforin) using the same cut-offs as for single gene analyses were performed and resulted in a higher accuracy (76.8%) for the diagnosis of acute rejection (P<0.001).

### Time of rejection diagnosis and effects of anti-rejection therapy

Acute rejection was clinically diagnosed in a mean of 9.3 days post-transplantation (range: 7 to 13 days) and the mean time for the molecular diagnosis was 5.3 days (range: 4 to 7 days) (P<0.01). Messenger RNAs expressions were compared at pre- and post-rejection treatment samples. TIM-3 gene median expression dropped 77.1% in the comparison of expression pre- and post-treatment for acute rejection (P=0.001). The drop of the perforin gene expression reached 90.9% in the same comparison (P=0.001).

## Discussion

In the present study, we evaluated mRNA expression for the diagnosis of acute rejection of kidney allografts. We found elevated expression of TIM-3 and perforin in patients with acute rejection that anticipated graft dysfunction. An elevated negative predictive value of gene expression analysis for the rejection diagnosis was also observed. However, considerable variation in mRNA expression occurred outside of the rejection episodes.

In the last decade, molecular techniques have been evaluated for the non-invasive diagnosis of renal allograft dysfunction, mainly for the detection of acute rejection, with analyses performed in either peripheral blood or urine (7,9,10). The non-invasive transcriptional approach has been developed in an attempt to avoid the need for allograft biopsy and better monitor the occurrence of graft injuries. However, the vast majority of the studies are cross-sectional and thus do not provide sequential graft evaluation and gene expression profile over time.

A number of different genes have been evaluated either in the peripheral blood (7,17,18) or in the urine (8,9,12,19,20) and have been shown to be well correlated in both and with tissue (10,13). Here, we report the longitudinal expression of the well-validated molecules perforin and TIM-3, both expressed by cytotoxic T lymphocytes (CTLs) that are activated during graft rejection (21,22). Perforin is stored in cytoplasmic granules and subsequently secreted by effector CTL leading to pore formation in the target-cell membrane, ultimately leading to cell death (14). Importantly, increased amounts of perforin protein have been demonstrated by immunostaining in human renal grafts undergoing acute rejection in previous studies with biopsy samples (23) and fine-needle aspirates (24). TIM-3 is a type I membrane protein preferentially expressed on terminally differentiated Th1 cells that seems to be central in the mechanisms of allograft rejection (15). TIM-3 has been associated with autoimmune diseases, tolerance induction, and to the regulation of Th1 immune responses (25,26). Previous studies have shown that perforin (7,8,10,17) and TIM-3 (12,13) mRNA expression in non-invasive cell samples (peripheral blood and urinary sediment cells) are augmented during acute rejection episodes of kidney grafts. TIM-3 protein expression has not been demonstrated in biopsies of renal transplant recipients. However, increased urinary concentration of soluble TIM-3 was demonstrated by ELISA by Chen and co-workers, in renal transplant recipients with acute rejection (27).

There is a paucity of longitudinal studies profiling non-invasive molecular biomarkers in kidney transplantation aiming at the diagnosis of acute rejection. In the present study, we observed increased mRNA expression of the perforin and TIM-3 genes in peripheral blood samples of patients that underwent acute renal graft rejection. Similarly, in the first reported longitudinal study, Simon and collaborators reported that acute cellular rejection could be detected by serial peripheral blood analyses of the perforin and granzyme B increased expression (17). Later, Suthanthiran et al. profiled urinary cell mRNA in the most robust longitudinal study available and have shown that a three-gene signature (18S ribosomal, CD3e mRNA, and interferon-inducible protein 10) discriminated acute cellular rejection from other causes of graft dysfunction (19). From these studies, it is possible to infer that increased mRNA expression of genes involved in the cytolytic attack occurs during acute cellular rejection as demonstrated at the available cross-sectional studies. Also, these studies have all found increased signaling before the clinical diagnosis of rejection. Suthanthiran et al. found that diagnostic gene signature precedes by 20 days the histological diagnosis of graft rejection (19) and Simon et al. described increased gene expression in a median of eleven days before the clinical diagnosis of rejection (17). The KSORT study was conceived to develop a test using a simple blood gene expression assay to detect patients at high risk for AR by using novel reference-based algorithm, using a 13 gene model set. In the KSORT study, although not longitudinally designed, it was also possible to anticipate AR up to three months prior to detection by renal biopsy taken at graft dysfunction (11). Accordingly, in the present study, increased gene expression was also detected before clinical diagnosis.

As in the previous longitudinal studies, borderline rejections were not included in the rejection group (17,19). Supporting this approach, previous high throughput molecular profiling studies have convincingly shown that a high proportion of borderline lesions do not carry a molecular signature of acute rejection and therefore are not attributable to rejection processes (28,29). Similarly, in the present study, the analysis of the expression levels of perforin and TIM-3 in patients with borderline rejection did not exhibit an acute rejection profile.

Another important finding of the present study is the down-regulation of mRNA expression observed in response to rejection treatment. Both perforin and TIM-3 mRNA transcripts decreased significantly upon rejection therapy. Similar results were described in cross-sectional studies, evaluating mRNA expression either in renal tissue (30) or in peripheral blood leukocytes (7). Also, in the longitudinal study by Simon et al., it was found that the expression of perforin and granzyme B decreased significantly after rejection treatment (17). Suthanthiran et al. reported that the score of the diagnostic signature for rejection decreased significantly following acute cellular rejection therapy. Interestingly, no association was found with the Banff grade for acute cellular rejection (19). Molecular features are suppressed by treatment more quickly than histopathology lesions, suggesting that they reflect the suppression of graft injury mechanisms better than histopathologic lesions, which can last longer, despite successful treatment (31). Taken together, these findings suggest that the non-invasive gene expression evaluations might become useful in monitoring the efficacy of immunosuppressive treatment of acute rejection.

Some dissimilarities were observed in the analysis of the diagnostic parameters derived from gene expression evaluation. Simon et al. reported that the positive predictive value (PPV) at initial times after transplantation seems to increase overtime (16). Suthanthiran et al. described sensitivity and specificity around 80% (19). In the present study, the diagnostic accuracy was lower than those reported in the previous longitudinal studies. Furthermore, accuracy was substantially lower than that observed in previous cross-sectional surveys. Importantly, and in accordance to Reeve et al., the negative predictive values were elevated indicating that rejection episodes would hardly occur in the absence of increased gene expression (32). The low PPV found in longitudinal studies suggests that high PPV found in cross-sectional studies might be misleading. Accordingly, the study by Simon et al. also reported PPVs that, although higher than those observed in the present study, were substantially lower than those reported in the cross-sectional studies. Also, in their study, the PPVs were increased by their approach to data reporting, which consisted of separating per period after transplantation and had different cutoff values for each time interval, leading to optimized but perhaps less clinically applicable results (16).

The reasons why prevalence studies show higher accuracy may be related to their strategy in evaluating cases and controls. These studies use a well-defined clinical phenotype (e.g. acute rejection), which is comparable to other equally well-defined situations, all retrospectively diagnosed. In this approach, overtime variations of gene expression are not detected. On the other hand, when samples are collected sequentially, variations in gene expression will be detected and will necessarily decrease the test accuracy. For instance, viral infections, including BK polyoma virus, which may be clinically silent, can elicit a TH1 response that involves many of the genes that also participate in the acute rejection phenomena (32). Subclinical rejection will not be detected unless sequential protocol biopsies are performed (33–35). Another possible reason for the false-positive results is the power of the molecular tool. It is conceivable that the RT-PCR detects very small, and perhaps not relevant, increments in gene expression.

The present study had weaknesses to be mentioned. Initially, gene selection for analyses was chosen from hypothesis-driven cross-sectional studies. Secondly, pre-transplant samples were not obtained and perhaps would be the ideal for comparisons of the subsequent samples. Thirdly, we did not perform protocol biopsies that could reveal subclinical aggressions and explain increased molecular expressions. Moreover, follow-up was restricted to three months. Finally, the study included a limited number of patients and no episodes of antibody-mediated acute rejection occurred in the study sample. In spite of the above, we believe that our study brought up and reinforced relevant findings such as the high negative predictive value and the effectiveness of the molecular approach for early diagnosis of acute rejection.

In conclusion, we suggest that the molecular non-invasive diagnosis of renal graft dysfunction, although currently not validated, is an approach with potential clinical usefulness and worth developing for future use.
